# Solid-state fermentation of palm kernels by *Yarrowia lipolytica* modulates the aroma of palm kernel oil

**DOI:** 10.1038/s41598-019-39252-9

**Published:** 2019-02-22

**Authors:** Wencan Zhang, Feifei Zhao, Fangju Zhao, Tiankui Yang, Shaoquan Liu

**Affiliations:** 1 Food Science and Technology Programme, Department of Chemistry, National University of Singapore, Science Drive 3, Singapore, 117543 Singapore; 2 Wilmar (Shanghai) Biotechnology Research & Development Center Co., Ltd, No. 118 Gaodong Road, Pudong New District, Shanghai, 200137 China; 3 National University of Singapore (Suzhou) Research Institute, No. 377 Linquan Street, Suzhou Industrial Park, Suzhou, Jiangsu 215123 China

## Abstract

Solid-state fermentation with *Yarrowia lipolytica* was applied to palm kernels (PK) with the aim to modulate the aroma of palm kernel oil (PKO) obtained after kernel roasting. The results showed that, the metabolic activities of *Y. lipolityca* brought about significant changes to the volatile profile of obtained PKO either by providing thermal reaction reactants or by directly contributing aroma compounds. After fermentation, a decreased content in glucose (60%) while an elevated amount (7-fold) in free amino acids was found in PK, which further impacted the formation of volatile compounds by influencing the Maillard reaction and Strecker degradation during roasting. More Strecker aldehydes and *N*-heterocyclic compounds were formed in PKO derived from fermented PK especially after intensified roasting. In addition, the catabolism of *Y. lipolytica* imparted some distinct volatile compounds such as 2-phenylethanol to the obtained PKO. However, the lipase excreted by *Y. lipolytica* hydrolysed PK lipids and released 5-fold more free fatty acids in fermented PKO, relative to the blank and control PKO, which likely contributed to the off-flavor. On the basis of all volatile categories, principal component analysis (PCA) clearly separated the fermented PKO from the blank and control PKO, with light roasted, fermented PKO being correlated with acids, alcohols and aliphatic aldehydes; medium and dark roasted, fermented PKO tending to be dominated by pyrroles, pyrazines and furanones, which is in correspondence with sensory changes of PKO. This study demonstrated that combining fermentation with roasting could provide a novel way to modulate the volatile composition and aroma of PKO.

## Introduction

Palm kernel oil (PKO) is the oil extracted from the seeds of oil palm (*Elaeis guineensis* Jackqu), also known as palm kernels (PK)^1^. Being one of the main vegetable oils, it is widely applied in food areas, whereby the aroma is a concern that affects its utilization directly^2^. The aroma of PKO is thought to be majorly formed during the roasting process, upon which thermal reactions such as Maillard reaction and caramelization occur^3,4^.

Our previous studies demonstrated that increasing the thermal reaction reactants such as free amino acids and reducing sugars in PK, by using either a commercial carbohydrase or a protease/peptidase can effectively modify the volatile profiles of roasted PK and the corresponding PKO^5–7^. Other than using commercial enzymes, fermentation is another natural approach that is expected to produce diverse aroma compounds and modify precursors, due to a wide array of extracellular enzymes especially hydrolases that could be produced by microorganisms^8,9^. Fermentation coupled with thermal reactions is one way by which the intense aroma in cocoa beans and coffee beans is generated^9,10^. It was reported that, without fermentation, roasted cocoa beans could not exhibit the typical cocoa flavor^11^. Furthermore, the aroma of roasted cocoa or coffee highly depends on the starter cultures applied, indicating the importance of fermentation in providing aroma precursors^12,13^.

*Y. lipolytica* is a strict aerobe and non-conventional dimorphic yeast that is generally recognized as safe (GRAS)^14^. It is present in different types of foods due to its nutritional versatility and physiological aspects, being distinguished for its capacity to secrete several enzymes to culture media: namely, two proteases (alkaline extracellular protease and acid extracellular protease), several lipases and phosphatases, a RNase and an esterase^15^. The strong proteolytic and lipolytic activities it exhibits influence the flavor development of sausage, cheese and meat due to the production of aroma compounds and precursors such as amino acids, fatty acids and esters^16–19^. In addition, *Y. lipolytica* has been successfully applied to ferment green coffee beans and okara (soybean residue) with the aim to improve the volatile profile^20–22^.

Given the fact that *Y. lipolytica* has the robust characteristics in providing aroma compounds and aroma precursors in various types of food substrates, it is expected that solid-state fermentation of PK by *Y. lipolytica* is able to module the volatile profile of PKO after kernel roasting, which has never been reported before. Thus, this study was conducted with the aim to evaluate the feasibility of *Y. lipolytica* fermentation for the aroma modification/enhancement of PKO.

## Materials and Methods

### Materials, Chemicals and Solvents

Fresh oil palm nuts were obtained from PT Wilmar Nabati, Indonesia. The palm nuts were a hybrid from Dura and Pisifera variety of *Elaeis guineensis* species harvest in 2016. After the hulls were removed, the PK, with a moisture content of 100 g/kg, was released.

The freeze-dried yeast culture, food grade *Y. lipolytica* NCYC 2904 was purchased from National Collection of Yeast Cultures (NCYC) (Norwich, UK). It was propagated in sterile yeast malt (YM) broth (2% glucose, 0.25% yeast extract, 0.25% bacteriological peptone and 0.2% malt extract, all w/v, pH 5.0) at 30 °C for 48 h under aerobic conditions to obtain a pure culture with a cell population of about 7 log CFU/mL. Glycerol was added to the pure culture at 15% v/v and it was stored at −80 °C before use.

Standards of sugars including arabinose, xylose, glucose, fructose, mannose, galactose, maltose and sucrose were purchased from Sigma Aldrich (St Louis, MO, USA). Standards of amino acids were obtained from Thermo Scientific (Rockford, IL, USA). Acetonitrile of HPLC grade was acquired from Tedia (Fairfield, OH, USA) and petroleum ether of ACS grade (boiling point of 35–60 °C) and ethanol were purchased from Merck (Darmstadt, Germany). AccQ-Fluor reagent kits and AccQ-Tag eluent A concentrate for amino acids analysis were provided by Waters (Dublin, Ireland).

### Solid-State Fermentation of Palm Kernels

Thawed pure yeast cultures of *Y. lipolytica* was added to sterile YM broth at 1% v/v and sub-cultured twice under the aforementioned conditions (*section 2.1*) to obtain a cell count of about 7 log CFU/mL. *Y. lipolytica* cells were obtained via centrifugation at 8000 × *g* for 10 min at 4 °C. The obtained cells were washed twice with 10% v/v phosphate saline buffer (pH 7.4) and the washed cells were resuspended in the same buffer to obtain the yeast preculture.

Whole fresh PK was crushed down into pieces (particle size 4–8 mm) by using a hammer. The crushed PK of 400 g was transferred to a glass container, to which 250 g deionized (DI) water was added and mixed with PK. The mixture was autoclaved at 121 °C for 15 min. The yeast preculture was added to the sterilized PK at 1% v/w (with the initial *Y. lipolytica* being approximate 5 log CFU/g of wet PK) and incubated at 20 °C for 72 h. Aerobic condition within the container was maintained by ensuring sufficient headspace (bed height: 4–5 cm; headspace: 3–4 cm) and by mixing the substrate bed every 12 h to introduce oxygen during sampling under aseptic conditions. Uninoculated, autoclaved PK incubated under the same conditions served as the control, while fresh, unheated PK served as the blank. All treatments were prepared in triplicate.

### Yeast Growth Determination

Sampling was conducted every 12 h for the determination of viable yeast cell counts. Four gram of PK were added to 36 mL of 0.1% w/v sterilized peptone water and the mixture was homogenized for 90 s. The homogenized mixture was appropriately diluted and plated on potato dextrose agar (PDA) plates, which were then incubated at 25 °C for 48 h before yeast enumeration. At least two samples were taken from each container to obtain an average count.

### Roasting of Palm Kernels

After 72 h incubation, the PK was oven-dried to a moisture content of 10% under 70 °C. The dried PK was then roasted in a baking oven for different times (10 min, 17 min and 24 min) under 180 °C to obtain light, medium and dark roasted levels, respectively.

### Determination of Moisture and Lightness of Palm Kernels

The moisture content of PK was measured by a moisture balance (Shimadzu MOC-120H, Kyoto, Japan), and the lightness (L* value, a representation of variation in the perception of a color or color space’s brightness) of PK was determined by a spectrophotometer (Konica Minolta CM-3500d, Osaka, Japan).

### Palm Kernel Oil Extraction

After roasting, PK was cooled to room temperature and transferred to a home-scale oil press (KM-022E, K.M. Electronic, Shanghai, China). Oil samples were collected and centrifuged for subsequent analysis.

### Analysis of Non-volatile Components in Palm Kernels

#### Soluble Sugars

PK powders with a uniform particle size were obtained after grinding and sifting through a No. 40 USA standard testing sieve (Fisher Scientific, Leicestershire, UK) before defatting. After defatting, 2.0 g of PK powder was extracted twice with 80% (v/v) ethanol and the extracts were combined and concentrated to 2 mL. Soluble sugars in the concentrated extract were analyzed by Shimadzu ultrafast liquid chromatography system (UFLC, Kyoto, Japan) coupled to a low temperature evaporative light scattering detector (ELSD) (Shimadzu, Kyoto, Japan). The detailed method used for soluble sugar extraction and analysis can be found in Zhang *et al*.^5^.

#### Free Amino Acids

1.0 g of defatted PK powder was used for free amino acids extraction by 1% trichloroacetic acid. Derivatization of the extracts was conducted by Waters AccQ-Tag system (Waters Corp., Waltham, MA, USA). Following derivatization, the extracts were separated by using Shimadzu UFLC (Kyoto, Japan) on a Waters AccQ-Tag Nova- Pak C18 column (150 × 3.9 mm, Waters, Dublin, Ireland) and detected by photodiode array detector (PDA). The detailed methodology used for free amino acid analysis is described in Zhang *et al*.^5^.

#### Volatile Compounds

Volatiles in 3.0 g of PKO were extracted and concentrated using headspace (HS)-solid phase microextraction (SPME) at 60 °C for 40 min and analyzed by gas chromatography (GC) coupled to flame ionization detector (FID) and mass spectrometer (MS) (Agilent, CA, USA). Methods for separation and identification of volatile compounds were the same as described in Zhang *et al*.^4^.

#### Sensory Evaluation

Sensory evaluation of PKO was carried out at Wilmar (Shanghai) Biotechnology Research & Development Centre, China. It was approved by the committee of Wilmar (Shanghai) R&D Center according to their research regulations. An experienced panel of 6 members (3 males and 3 females, between the age of 20 and 40) participated in sensory evaluation and informed consent was obtained from all participants. Quantitative descriptive analysis was used to evaluate the sensory property. The detailed method used for sensory evaluation can be found in Zhang *et al*.^5^.

#### Statistical Analysis

All experiments were performed in triplicate. The values of all parameters were examined by one-way ANOVA. Figures were created using Graphpad Prism 5. Principal component analysis (PCA) was performed using SPSS 19.0.

## Results and Discussion

### Yeast Population

**Figure 1:**
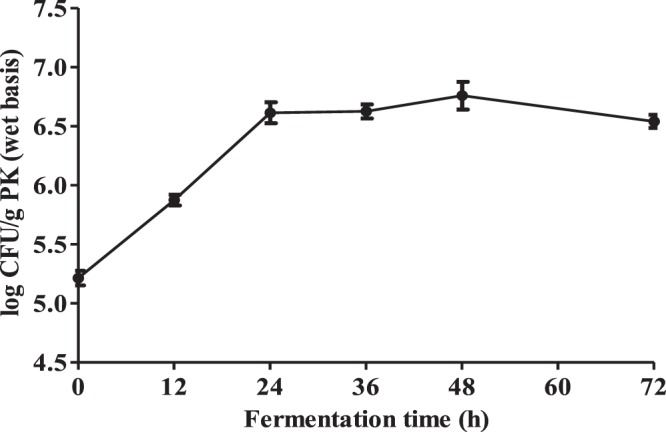
Time-course changes in viable yeast cell counts. Results are presented as mean values ± SD (n = 3).

The viable yeast cell count increased from 5.21 to 6.61 log CFU/g wet PK in the first 24 h, and remained relatively stable thereafter with a final cell count of 6.5 log CFU/g wet PK at 72 h, as can be seen from Fig. 1. This indicated that the autoclaved PK supported the growth of *Y. lipolytica* in solid-state fermentation setup without any nutritional supplementation.

### Moisture Content and Lightness (L*) of Palm Kernels

After different pretreatments, the obtained PKs (blank, control and fermented) were oven-dried and roasted. PK samples with different roast degrees were collected and the moisture content and L* value were determined to differentiate the roast levels; the results are provided in Table 1.

**Table 1:** Moisture content (mg/g) and L* value of blank, control and fermented palm kernels (PK) with different roast degrees.

	Moisture of PK (mg/g)^A^			L* value of PK		
	Blank	Control	Fermented	Blank	Control	Fermented
Before roasting	103.9 ± 10.7^a^	108.9 ± 14.5^a^	110.9 ± 15.7^a^	51.26 ± 4.68^a^	47.93 ± 0.24^a^	45.61 ± 0.18^a^
Light roast	49.7 ± 3.0^a^	32.5 ± 5.0^b^	51.9 ± 1.0^a^	30.23 ± 0.39^a^	31.28 ± 1.41^a^	38.23 ± 0.87^b^
Medium roast	23.7 ± 0.4^a^	17.6 ± 2.8^b^	23.0 ± 1.9^a^	25.31 ± 0.45^a^	26.91 ± 0.21^a^	30.46 ± 1.32^b^
Dark roast	3.6 ± 3.1^a^	5.4 ± 3.2^a^	6.1 ± 1.4^a^	23.96 ± 0.41^a^	24.40 ± 0.41^a^	25.19 ± 0.24^a^

^A^Results are presented as mean values ± SD (n = 3). Mean values with different letters in the same row indicate significant differences among blank, control and fermented PK at the same roast degree (*p* < 0.05).

As can be seen from Table 1, the moisture content dropped significantly from ≈100 mg/g to roughly 5 mg/g upon roasting, and the corresponding L* value decreased from approximate 50 to 24 for the blank, control and fermented PK after dark roast.

In comparison, there is no significant difference in both moisture content and L* value among the blank, control and fermented PK before roasting. However, after light and medium roast, lower moisture was found in the control PK than blank and fermented PK. Meanwhile, the fermented PK had a brighter color (higher L* value), indicating weaker non-enzymatic browning occurred, likely due to the consumption of sugars by the yeast. Nevertheless, after dark roast, the difference in either moisture content or L* value among the blank, control and fermented PK disappeared.

### Non-Volatile Compounds in Palm Kernels with Various Roast Degrees

#### Soluble Sugars

The content of soluble sugars in the blank, control and fermented PK subjected to various roast degrees was determined and the results are presented in Table 2. When comparing the sugar contents in the control PK to that in the blank PK, it was found that autoclaving had induced some changes to the sugar profile with higher amounts being noticed in fructose and glucose in the control PK, respectively, which might be due to the thermal degradation of polysaccharides during autoclaving.

**Table 2:** Changes of soluble sugars in blank, control and fermented palm kernels (PK) with different roast degrees.

	Concentration (mg/g dry matter) in defatted kernels^A^											
	Before roasting			Light roast			Medium roast			Dark roast		
	Blank	Control	Fermented	Blank	Control	Fermented	Blank	Control	Fermented	Blank	Control	Fermented
Xylose	—^B^	—	—	0.61 ± 0.06^a^	0.56 ± 0.07^a^	0.32 ± 0.06^b^	—	0.32 ± 0.03^a^	0.40 ± 0.03^a^	—	—	—
Arabinose	—	—	—	0.36 ± 0.01^a^	0.33 ± 0.02^a^	—	—	—	0.34 ± 0.02^a^	—	—	—
Fructose	2.58 ± 0.26^a^	4.20 ± 0.30^b^	3.55 ± 0.18^b^	1.73 ± 0.06^a^	2.24 ± 0.26^a^	2.08 ± 0.40^a^	0.43 ± 0.02^a^	0.67 ± 0.20^ab^	0.93 ± 0.11^b^	0.34 ± 0.01^a^	0.29 ± 0.01^a^	0.31 ± 0.00^a^
Glucose	2.51 ± 0.32^a^	3.45 ± 0.19^b^	1.52 ± 0.47^c^	1.39 ± 0.05^a^	1.52 ± 0.13^a^	0.95 ± 0.12^b^	0.39 ± 0.23^a^	—	1.04 ± 0.21^b^	—	—	—
Sucrose	14.58 ± 4.04^a^	15.01 ± 2.20^a^	15.39 ± 5.50^a^	2.99 ± 0.12^a^	10.66 ± 1.11^b^	15.76 ± 1.34^c^	0.65 ± 0.09^a^	1.51 ± 0.41^a^	3.60 ± 0.71^b^	0.62 ± 0.13^a^	0.49 ± 0.04^a^	—
Mannose	—	—	1.29 ± 0.32^a^	—	—	0.86 ± 0.41^a^	—	—	—	—	—	—
Galactose	2.22 ± 0.21^a^	3.64 ± 0.41^a^	2.12 ± 1.07^a^	2.29 ± 0.15^a^	3.17 ± 0.71^a^	2.13 ± 0.24^a^	2.40 ± 0.50^ab^	3.17 ± 0.18^a^	1.41 ± 0.76^b^	3.07 ± 0.04^a^	2.78 ± 0.47^a^	3.36 ± 0.09^a^
Maltose	—	—	—	0.89 ± 0.12^a^	1.44 ± 0.27^a^	1.86 ± 0.80^a^	0.58 ± 0.30^a^	—	0.61 ± 0.08^a^	—	0.73 ± 0.07^a^	0.40 ± 0.09^b^
Total	21.88 ± 4.54^a^	26.29 ± 1.56^a^	23.87 ± 6.70^a^	10.25 ± 0.04^a^	19.91 ± 2.28^b^	23.96 ± 1.68^b^	4.45 ± 0.47^a^	5.67 ± 0.82^ab^	8.34 ± 1.81^b^	4.03 ± 0.17^a^	4.30 ± 0.56^a^	4.07 ± 0.06^a^

^A^Results are presented as mean values ± SD (n = 3). Mean values with different letters indicate statistical differences among blank, control and fermented PK with the same roast degree (*p* < 0.05).

^B^“—” = Undetected.

After fermentation, an obvious decline of 60% in glucose was observed as compared with the control. This is probably due to the utilization of glucose to produce compounds such as organic acids, lipids, pyruvate, and enzymes, among others, by *Y. lipolytica*^23,24^. In comparison, fructose was not used by *Y. lipolytica* as no significant change was observed. This observation was in accordance with a previous finding which showed that fructose is only used after any available glucose has been completely consumed due to the fact that the *Y. lipolytica* hexokinase has a low affinity for fructose^25,26^. Similarly, sucrose, which remained unchanged after fermentation, also cannot be utilized by *Y. lipolytica* because of the lack of the sucrose-cleaving enzyme invertase^27^. Meanwhile, mannose, which was absent in the control and blank PK, was released after fermentation. The ability to release mannose by *Y. lipolytica* was probably due to the secreted mannosidases. An exo-α-mannosidase was characterized in *Y. lipolytica*^28^.

After light roast, small amounts of xylose, arabinose and maltose (all of which was not found before roasting) were released in PKs (blank, control and/or fermented), probably due to the thermal breakdown of polysaccharides; they were subsequently consumed when roast degree proceeded. This is also true for fructose, glucose, sucrose and mannose, the concentration of which declined clearly after different degrees of roasting. As key Maillard reaction and caramelization reactants, they most probably participated in these thermal reactions which further contributed to the generation of aroma compounds^29,30^.

It is worth noting that, the decline of simple sugars in the fermented PK was relatively slower than that in the blank and control PK with light roast, indicating fermentation had impacted the thermal reactions in PK upon roasting by delaying the sugar consumption.

#### Free Amino Acids

*Y. lipolytica* is known to possess proteolytic activities, thus the content of free amino acids in the blank, control and fermented PK was determined, the results of which are presented in Table 3.

**Table 3:** Changes of free amino acids in blank, control and fermented palm kernels (PK) with different roast degrees.

	Concentration (mg/kg dry matter) in defatted kernels^A^											
	Before roasting			Light roast			Medium roast			Dark roast		
	Blank	Control	Fermented	Blank	Control	Fermented	Blank	Control	Fermented	Blank	Control	Fermented
Asp	—^B^	—	135.77 ± 36.25^a^	—	—	—	—	—	—	—	—	—
Ser & Asn	505.27 ± 71.26^a^	—	205.50 ± 50.37^b^	—	—	—	—	—	—	—	—	—
Glu	342.69 ± 23.85^a^	411.59 ± 76.96^a^	349.81 ± 95.29^a^	—	—	—	—	—	—	—	—	—
Gly	9.93 ± 0.66^a^	11.40 ± 5.08^a^	200.79 ± 53.11^b^	2.74 ± 0.47^a^	—	88.15 ± 10.51^b^	—	9.52 ± 0.73^a^	16.01 ± 4.15^a^	—	—	—
His & Gln	—	31.58 ± 12.20^a^	765.23 ± 242.62^b^	13.09 ± 4.56^a^	—	167.88 ± 12.33^b^	10.04 ± 1.79^a^	—	93.39 ± 11.30^b^	—	—	48.87 ± 1.85^a^
Arg	86.12 ± 10.90^a^	125.50 ± 60.06^a^	294.81 ± 62.97^b^	—	—	60.96 ± 20.63^a^	—	—	—	—	—	—
Thr	16.74 ± 0.19^a^	30.91 ± 9.45^a^	288.00 ± 82.22^b^	—	—	65.06 ± 2.34^a^	—	—	—	—	—	—
Ala	37.54 ± 5.75^a^	68.25 ± 25.78^a^	489.50 ± 120.86^b^	12.02 ± 6.82^a^	22.85 ± 10.13^a^	153.41 ± 16.05^b^	5.45 ± 0.77^a^	7.12 ± 1.69^a^	56.11 ± 10.80^b^	—	—	15.28 ± 0.89^a^
Pro	17.83 ± 1.87^a^	30.54 ± 9.88^a^	267.00 ± 74.27^b^	5.35 ± 2.21^a^	—	76.24 ± 6.19^b^	—	—	—	—	—	—
Cys	—	—	52.76 ± 13.89^a^	8.96 ± 4.21^a^	—	—	8.34 ± 3.60^a^	—	—	—	6.40 ± 0.28^a^	—
Tyr	—	—	147.54 ± 57.66^a^	—	—	51.08 ± 11.67^a^	—	—	—	—	—	—
Val	14.23 ± 1.77^a^	—	412.98 ± 141.34^b^	—	—	38.38 ± 9.60^a^	—	—	—	—	—	—
Met	—	—	445.27 ± 151.33^a^	—	—	146.50 ± 7.78^a^	—	—	—	—	—	—
Ile	10.56 ± 0.70^a^	—	262.06 ± 78.85^b^	—	—	64.92 ± 3.11^a^	—	—	—	—	—	—
Leu	9.66 ± 0.51^a^	—	469.54 ± 147.18^b^	—	—	92.09 ± 5.84^a^	—	—	—	—	—	—
Phe	13.38 ± 0.14^a^	—	334.29 ± 104.73^b^	—	—	77.02 ± 14.80^a^	—	—	—	—	—	—
Try	16.40 ± 0.19^a^	—	53.19 ± 15.26^b^	—	—	—	—	—	—	—	—	—
Total	1,084.28 ± 118.31^a^	706.33 ± 89.56^a^	5,188.91 ± 1,527.92^b^	42.16 ± 13.44^a^	22.85 ± 10.13^a^	1,081.70 ± 33.38^b^	23.83 ± 6.10^a^	16.65 ± 1.91^a^	165.52 ± 26.00^b^	0	6.40 ± 0.28^a^	64.14 ± 2.64^b^
NH_3_	4.27 ± 0.66^a^	7.09 ± 3.26^ab^	14.86 ± 4.26^b^	4.68 ± 3.37^a^	2.65 ± 2.37^a^	4.55 ± 1.04^a^	2.68 ± 1.09^a^	2.47 ± 0.14^a^	5.54 ± 1.09^a^	—	—	—

^A^Results are presented as mean values ± SD (n = 3). Mean values with different letters indicate statistical differences among blank, control and fermented PK with the same roast degree (*p* < 0.05).

^B^“—” = Undetected.

From Table 3 we can see that, the major amino acids found in the natural PK (blank) were glutamic acid, co-eluted serine and asparagine, as well as arginine. The result was consistent with a previous study on the amino acid composition of palm kernel meal, which found that glutamic acid and arginine were the most abundant (16.8 g/16 g of N and 11.56 g/16 g of N, respectively)^31^.

After autoclaving, some free amino acids in the control PK became undetectable such as valine, isoleucine, leucine, phenylalanine and tryptophan. This is expected since Maillard reaction or thermal degradation would have occurred during autoclaving, which had consumed some free amino acids.

When comparing the free amino acids content in the fermented PK to that in the control PK, a remarkable increment in concentration was found in almost all individual amino acids after *Y. lipolytica* fermentation; the total free amino acid concentration has increased by 7-fold. This increment in amino acids highlights the strong proteolytic capability of *Y. lipolytica*, which produces both extracellular acid and alkaline proteases^32^. Releasing free amino acids by *Y. lipolytica* was also found in okara and cheese fermentation, and this is essential for cheese ripening^22,33^.

After roasting, the content of all individual amino acids decreased obviously. This was expected since amino acids are essential reactants in Maillard reaction and Strecker degradation, whereby there are consumed and partly converted into *N*-containing volatile compounds.

### Changes in Volatile Compounds in Palm Kernel Oil

#### Changes in Overall Volatile Profiles

**Figure 2:**
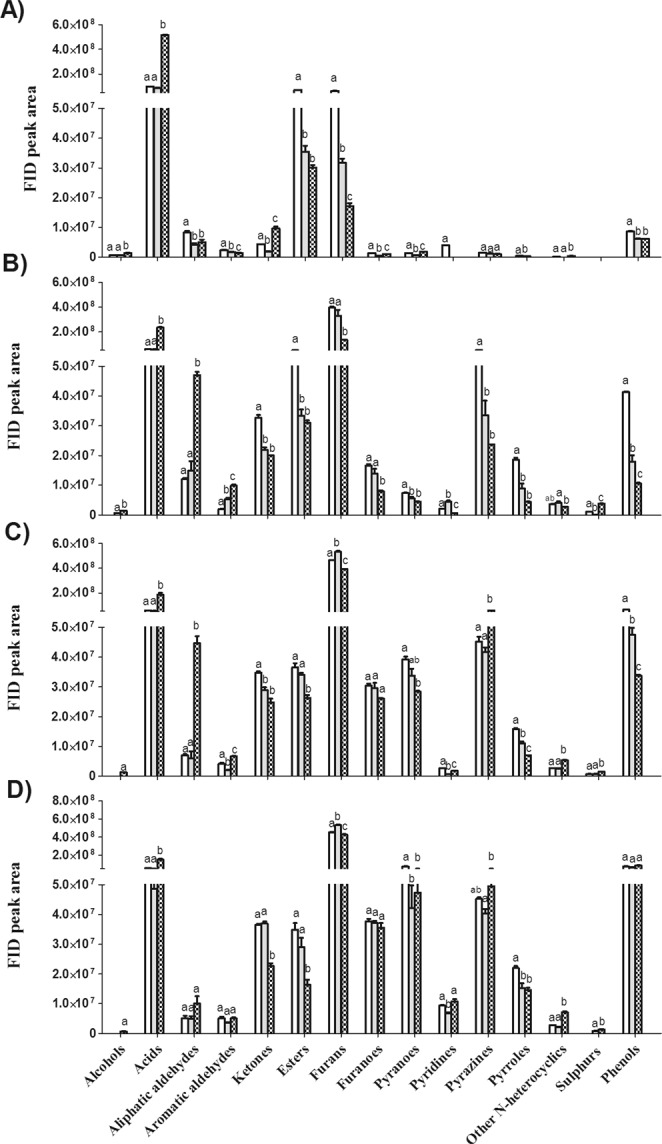
Changes in FID peak area of overall volatile profiles in palm kernel oil (PKO) derived from blank (), control () and fermented palm kernels (PK) () with different roast degrees (**A**, before roasting; **B**, light roast; **C**, medium roast; **D**, dark roast). Columns with different letters indicate statistical differences among PKOs with the same roast degree (*p* < 0.05). Results are presented as mean values ± SD (n = 3).

The volatile compounds detected in PKO were categorized into 15 classes, which were alcohols, acids, aliphatic aldehydes, aromatic aldehydes, ketones, esters, furans, furanones, pyranones, pyridines, pyrazines, pyrroles, other *N*-heterocyclic compounds, sulphurs and phenols, according to their chemical structures and functional groups. The FID peak areas of each volatile class in PKO with different kernel roasting degrees are shown in Fig. 2.

The volatile profiles varied dramatically among PKOs with different pretreatments even without roasting (Fig. 2A). After fermentation, changes were found in almost all volatile categories, showing that the metabolic activities of *Y. lipolytica* has markedly influenced the volatile composition of PK and corresponding PKO; however, the pattern varied greatly among different volatile categories.

The most obvious change was found in volatile acids, whereby 5-fold more acids were found in the PKO produced from the fermented PK before roasting, as compared with that in the PKOs made from the blank and control PK; the higher level of acids in the fermented PKO persisted throughout the whole roasting process. Significant increases were also found in the content of aldehydes and alcohols in the fermented PKO before roasting and after roasting. On the other hand, decrease in furans was the most evident after fermentation and roasting. Furans are generally sugar breakdown products; the decline in furans in the fermented PKO implies that less sugars had been consumed and converted. Of the *N*-heterocyclics, their content decreased initially (before roasting and after light roast) and elevated when the roast degree was intensified (medium and dark roast). The most representative volatile compounds are discussed in detail in the following sections.

#### Changes in Volatile Acids

**Figure 3:**
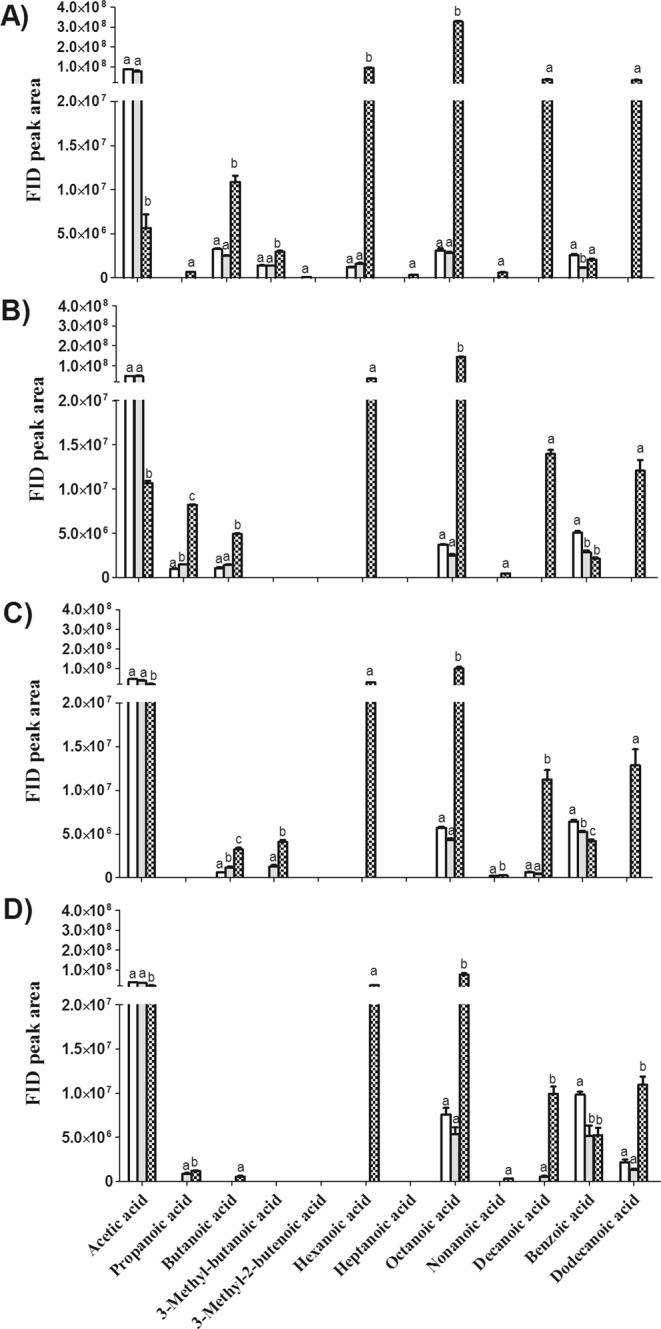
Changes in FID peak area of volatile acids in palm kernel oil (PKO) derived from blank (), control () and fermented palm kernels (PK) () with different roast degrees (**A**, before roasting; **B**, light roast; **C**, medium roast; **D**, dark roast). Columns with different letters indicate statistical differences among PKOs with the same roast degree (*p* < 0.05). Results are presented as mean values ± SD (n = 3).

As mentioned above, the change in volatile acids was the most dramatic after fermentation; the profiles of individual acids in PKO with different roast degrees are shown in Fig. 3.

One noticeable observation is the content of acetic acid, which declined by 14-fold in PKO immediately after fermentation relative to that of the control and blank PKO (Fig. 3A). Acetic acid might be consumed by the yeast during fermentation, as previous studies have shown that *Y. lipolytica* is able to use acetic acid as the sole carbon and energy source^34,35^. Meanwhile, remarkable increases were found in other free fatty acids, with butanoic, hexanoic, octanoic, decanoic and dodecanoic acids being the most representative. Besides proteases discussed above, *Y. lipolytica* is distinguished for its capacity to secrete several lipases, depending on media composition and environmental conditions; the extracellular lipases are able to hydrolyse triglycerides into free fatty acids and glycerol^36,37^. The lipolytic activity of *Y. lipolytica* is considered very important to the quality of sausage, since the produced free fatty acids and their degradation products such as methyl ketones are substrates responsible for typical aroma development^38^. In addition, the ability of *Y. lipolytica* to release free fatty acids in cheese ripening largely determines the flavor of some dairy products such as smear-ripened cheeses^39^. However, free fatty acids are generally undesirable in edible oils, as they can impart rancid, sweat, soapy and oily sensory attributes especially for the short-chain fatty acids.

After different levels of roasting, the amount of volatile acids declined to some extent, probably due to the evaporation and thermal degradation; however, they still represent a large portion of volatile compounds, which might impart unpleasant sensory notes to PKO.

#### Changes in Aldehydes

**Figure 4:**
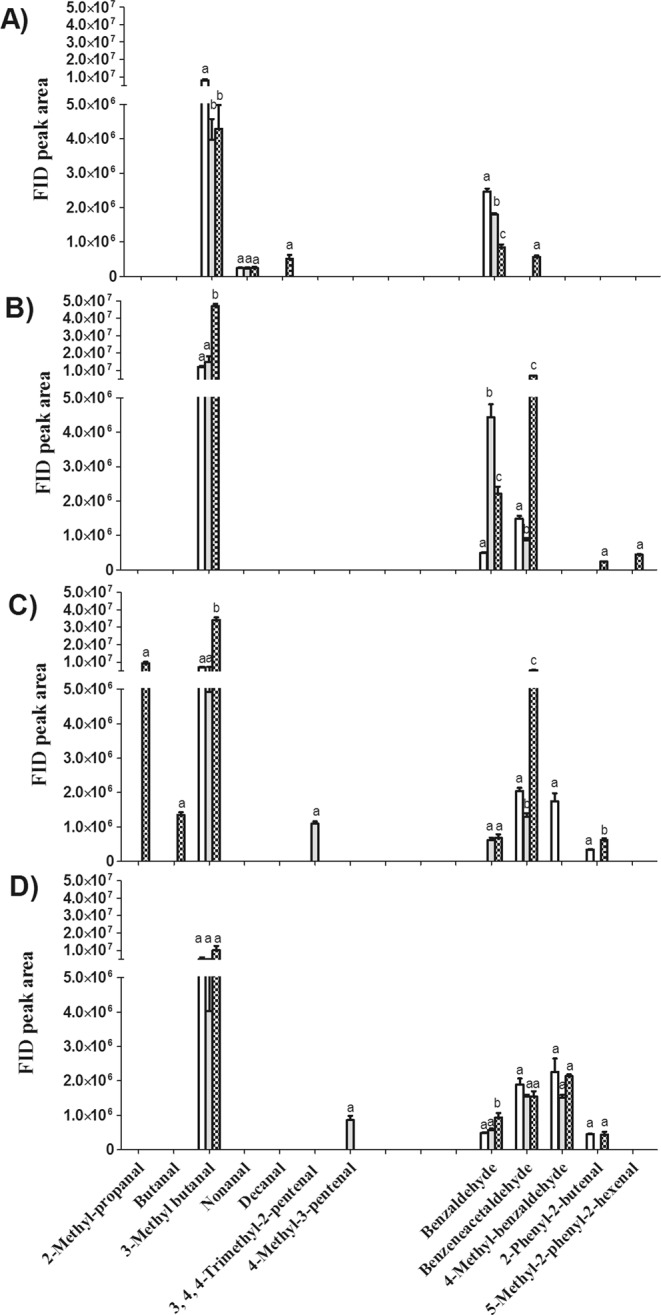
Changes in FID peak area of volatile aldehydes in palm kernel oil (PKO) derived from blank (), control () and fermented palm kernels (PK) () with different roast degrees (**A**, before roasting; **B**, light roast; **C**, medium roast; **D**, dark roast). Columns with different letters indicate statistical differences among PKOs with the same roast degree (*p* < 0.05). Results are presented as mean values ± SD (n = 3).

Changes in aldehydes in PKO with different pretreatments and with different roast degree were also remarkable, which are shown in Fig. 4.

After fermentation, decreases in benzaldehyde but increases in benzeneacetaldehyde were observed, as shown in Fig. 4A. A decline in benzaldehyde was also found in cheese agar when it was fermented with *Y. lipolytica* CBS 2075^40^. The boosted content of benzeneacetaldehyde might have resulted from the bioconversion of phenylalanine due to the catabolism of *Y. lipolytica*.

After roasting, obvious increases in benzeneacetaldehyde and 3-methyl butanal were found in PKO for light roast and medium roast. A high level of 2-methyl propanal was also found in the fermented PKO with medium roast. All of these compounds are Strecker aldehydes, originating from their amino acid precursors during heating. 3-Methyl butanal with a malt-like sensation could be formed through reactions between leucine and dicarbonyl compounds^41^. Similarly, benzeneacetaldehyde with a floral odor could be generated from its precursor phenylalanine whereas 2-methyl propanal with floral and malty odors might have originated from valine^41^. Higher levels of the aforementioned aldehydes found in fermented PKO might be due to the elevated contents of their respective amino acid precursors.

#### Changes in Alcohols

**Figure 5:**
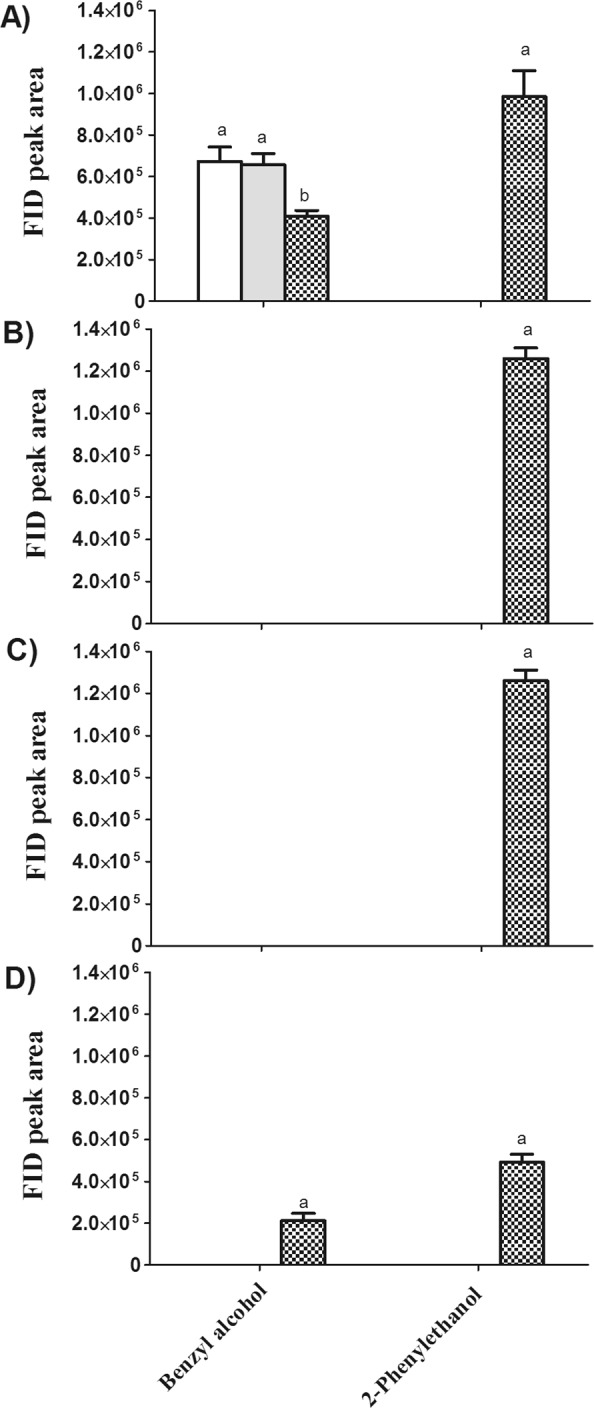
Changes in FID peak area of volatile alcohols in palm kernel oil (PKO) derived from blank (), control () and fermented palm kernels (PK) () with different roast degrees (**A**, before roasting; **B**, light roast; **C**, medium roast; **D**, dark roast). Columns with different letters indicate statistical differences among PKOs with the same roast degree (*p* < 0.05). Results are presented as mean values ± SD (n = 3).

As can be seen from Fig. 5, benzyl alcohol decreased after fermentation as alcohols can be the carbon source of *Y. lipolytica*^27^. It is interesting to note that, 2-phenylethanol was only found in PKO after fermentation. This finding was in accordance with previous research, in which *Y. lipolytica* was reported as the novel and promising 2-phenylethanol producer via bioconversion of L-phenylalanine^42^.

After roasting, benzyl alcohol disappeared, which is most probably due to the thermal breakdown or evaporation, whereas 2-phenylethanol is relatively thermally stable. With a rose-like flavor note, the consistent presence of 2-phenylethanol in fermented PKO with various roast degrees may impart a distinct sensory note to PKO.

#### Changes in *N*-heterocyclic Compounds

Pyrazines and pyrroles are predominant compounds in thermally processed food, if not the most, having been identified in various food products^43^. The sensory properties of these compounds are potent with low thresholds, contributing to the typical roasted and nutty flavor to the heated foods^43^.

**Figure 6:**
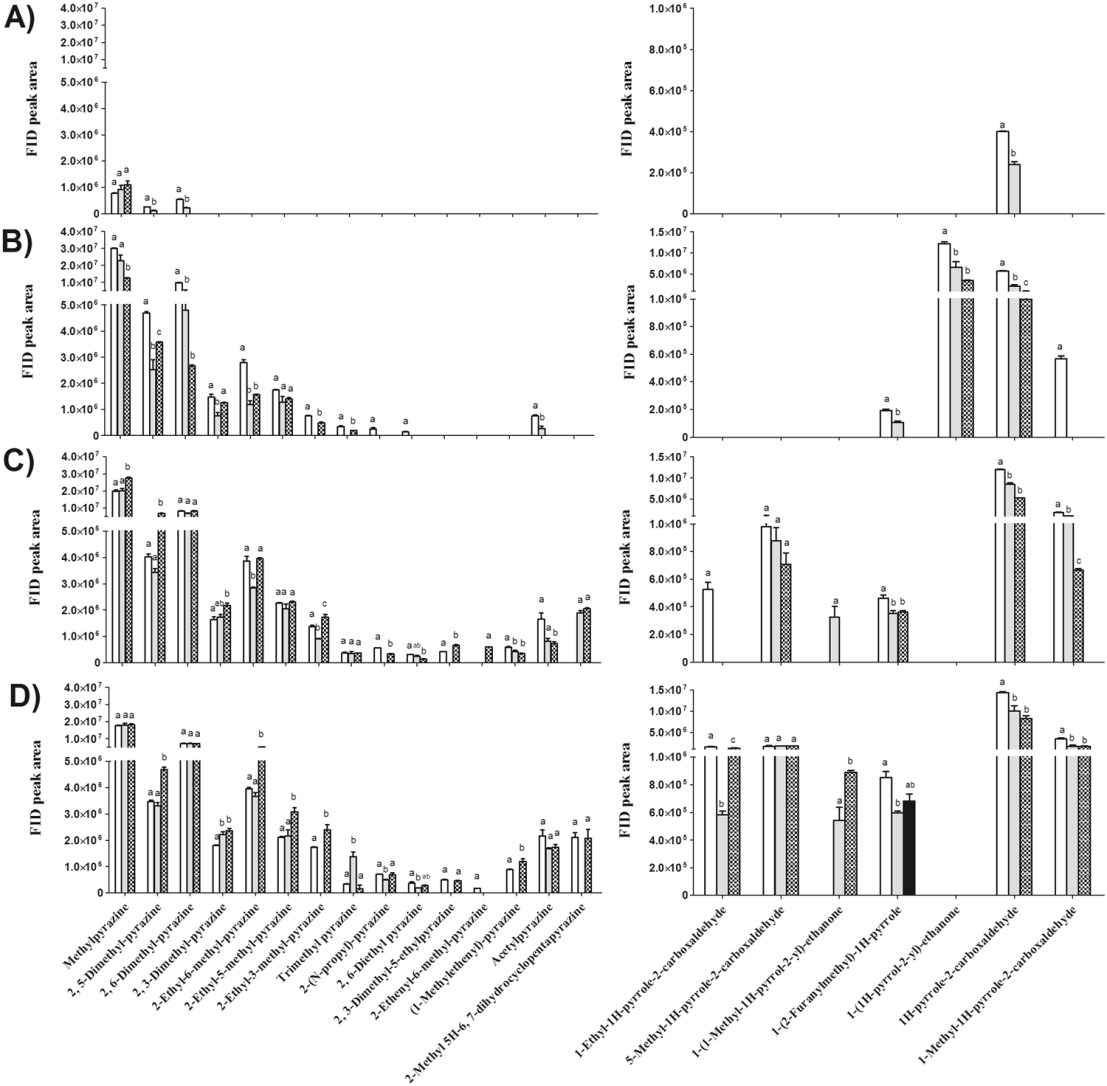
Changes in FID peak area of pyrazines (left) and pyrroles (right) in palm kernel oil (PKO) derived from blank (), control () and fermented palm kernels (PK) () with different roast degrees (**A**, before roasting; **B**, light roast; **C**, medium roast; **D**, dark roast). Columns with different letters indicate statistical differences among PKOs with the same roast degree (*p* < 0.05). Results are presented as mean values ± SD (n = 3).

From Fig. 6 it can be seen that fermentation did not bring about direct changes to either pyrazines or pyrroles, as very few such compounds were found before roasting and the difference between PKOs from fermented and unfermented PKs was slight, with the content of 2,5-dimethyl pyrazine and 2,6-dimethyl pyrazine and 1H-pyrrole-2-carboxaldehyde being different between them.

After roasting, the profile of pyrazines and pyrroles changed evidently in all PKOs with a remarkable increasing trend being found in all individual compounds with the progression of roasting. As both of them are produced from thermal reactions such as Strecker degradation, roasting accelerates their generation.

When comparing the content of pyrazines and pyrroles in the control PKO with that in the blank PKO, a slight decrease trend was found in some compounds in the control PKO. As autoclaving consumed some free amino acids, the generated pyrazines and pyrroles in the control PK were expected to be less than that of the blank.

It is known that the formation of pyrazines and pyrroles is highly correlated with free amino acids. For example, alkyl pyrazines are known to be formed from the reaction of α-diketones with amino acids via Strecker degradation^44^. However, even though much more free amino acids were found in the fermented PK, the contents of pyrazines and pyrroles found in fermented PKO were not correspondingly higher, especially at light roast level, which is unexpected.

One explanation would be that the release of free fatty acids and the possible synthesis of other non-volatile organic acids would have lowered the pH in the fermented PK hence mitigating the generation of pyrazines. Most steps in the Maillard reaction are sensitive to pH, and small changes in pH can alter the aroma profile^43^. It is generally known that pH has an impact on the generation of pyrazines, hindering their formation at low pH due to the protonation of the amino groups of the amino acids and peptides^45^.

Another explanation would be that the generation pathway of *N-*heterocyclics had been interfered by the participation of lipid oxidation products. A previous study found that the generation of pyrazines in xylose-lysine and glucose-lysine model systems appeared to be particularly sensitive to the presence of the vegetable oils such as olive, canola, and sunflower oils during thermal treatment, implying that the thermal oxidation products of these unsaturated fats may affect the generation of pyrazines, although the exact underlying mechanism was not provided by the authors^46^. In the current study, lipolysis by *Y. lipolytica* released more free fatty acids, which are more susceptible to oxidation than esterified fatty acids^44^. Under such a situation, more lipid oxidation products would have been produced in fermented PK and PKO than that in control and blank PK and PKO. The oxidation of lipids produces a variety of aldehydes that can participate in carbonyl-amine condensation and aldol condensations, effectively competing with reducing sugars and reactive intermediates in Maillard reaction for amino groups^44^. As such, the formation of pyrazines and pyrroles was negatively affected.

Nevertheless, after medium and dark roast, those *N*-heterocyclic compounds in fermented PKO caught up and even exceeded that in the control PKO, indicating the proteolytic activity of *Y. lipolytica* had ultimately impacted the formation of volatile compounds by fortifying Maillard reaction and Strecker degradation when the roast degree was advanced. Our previous study showed that 2,6-dimethyl pyrazine, 2,5-dimethyl pyrazine, 2,3-dimethyl pyrazine and 1-ethyl-1H-pyrrole-2-carboxaldehyde were all the aroma-active volatiles in PKO, all of which were found in heightened levels in fermented PKO^4^. Consequently, they would help to account for a different aroma profile of fermented PKO.

It should be noted that, autolysis of yeast during fermentation might have released some aroma precursors such as amino acids, peptides, lipids, polysaccharides, nucleotides and nucleosides, which would in turn impact the aroma composition in obtained PKO after roasting, however, in our view, to a low extent^47–49^. It is known that autolysis usually occurs at the end of stationary growth phase when the fermentable carbon sources are almost depleted and is normally associated with cell death^47,50^. In the current experiment, both amino acids and simple sugars were left with relatively high amount when fermentation was stopped, indicating that nutrients for yeast survive were available. According to the growth curve of *Y. lipolitica* in our preliminary experiment (data not shown), the stationary phase lasted at least for 4 days without obvious decrease in cell numbers. In the current study, fermentation was stopped at day 3 when the stationary phase was supposed to be ongoing.

In addition, the thermal lysis of yeast cells upon roasting may also have induced the generation of some aroma compounds such as furanthiols and pyrazines^51,52^. When heated, yeast cells are supposed to collapse and degrade eventually and fragments such as saccharides, proteins and lipids would participate in thermal reactions and interact with other ingredients, which will result in the generation of some aroma compounds. However, we believe this might not have contributed significantly to the final volatile profile in this study. It is known that sulfur-containing odorants, such as 5-methylfurfurylthiol, 2-methyl-3-furanthiol, 2,2-furfurylthiol or 3-mercapto-2-butanone, have been identified as the most important aroma compounds in thermally treated yeast extracts^52,53^, all of which were not found in our study (data not shown). In addition, thermal reactions of yeast extract give a typical meat-like and savory flavor, which was also not detected in sensory evaluation.

#### Principal Component Analysis (PCA) of the Volatile Profiles in Palm Kernel Oil

**Figure 7:**
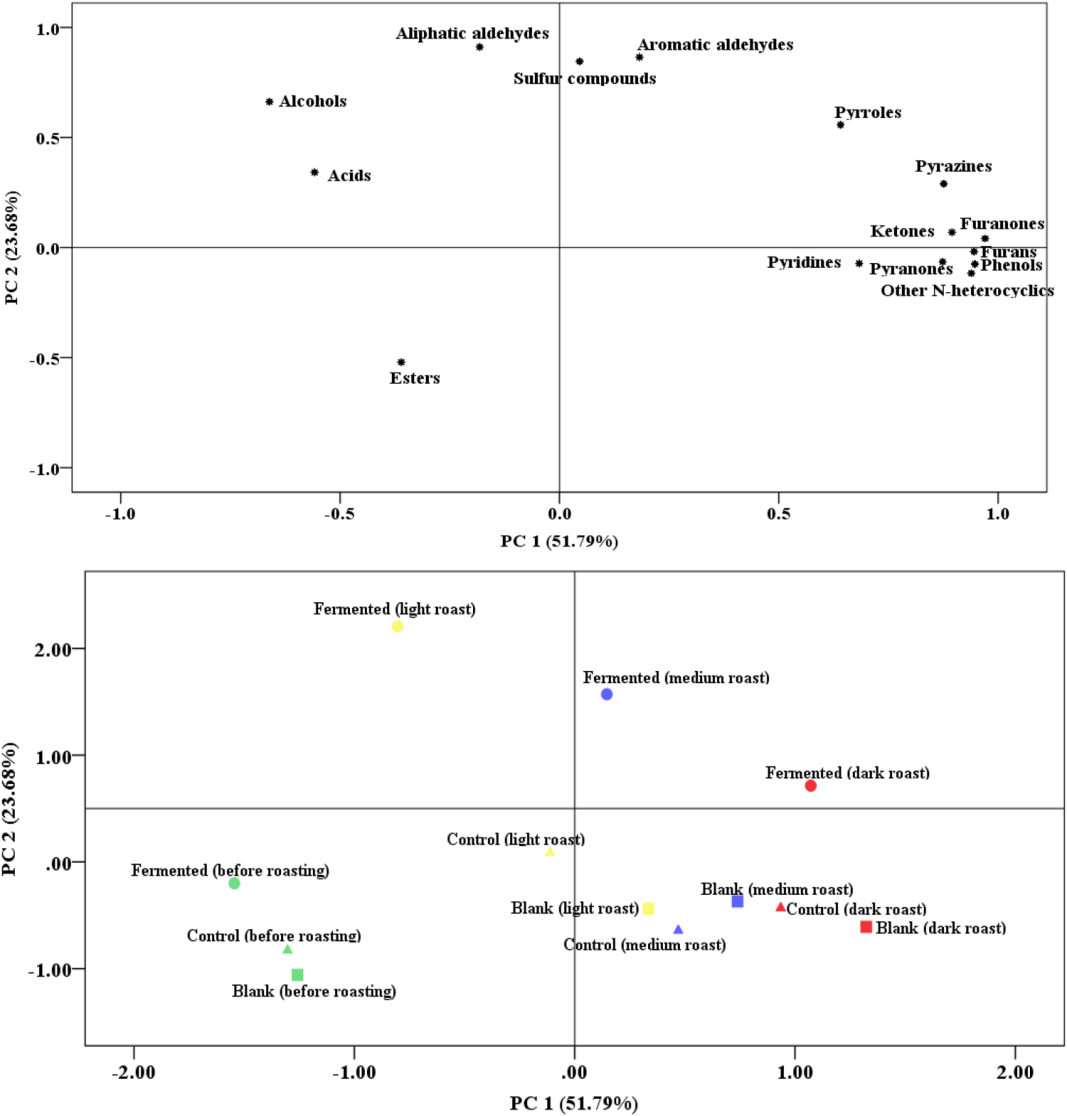
Loading plot (above) and score plot (below) of PCA for the volatile compounds in palm kernel oil (PKO) made from blank, control and fermented palm kernels (PK) with different roast degrees.

PCA was carried out on the basis of all volatile categories with the aim to gain a comprehensive comparison of the aroma profile of PKOs with different pretreatments and with different roast degrees. The first two PCs accounted for 75.47% of total variability in the dataset, with PC1 and PC2 accounting for 51.79% and 23.68%, respectively (Fig. 7). Before roasting, the three types of PKO shared a similarity, all of which were negatively correlated with PC1 and PC2. However, after roasting, PCA clearly discriminated the fermented PKO with various kernel roasting degrees from the control and blank PKO; they differed from each other by having an opposite direction at PC2 (Fig. 7). The profiles of control and blank PKO with various roast degrees are similar to each other. Specifically, the PKO from light roasted and fermented PK was more correlated with acids, alcohols and aliphatic aldehydes. After medium and dark roast, fermented PKO tended to be dominated by aromatic aldehydes, pyrroles, pyrazines and furanones. This indicates that, PKO made from fermented PK after different levels of roasting represents a different volatile profile as compared to the control and blank PKO.

#### Sensory Evaluation of Palm Kernel Oil

**Figure 8:**
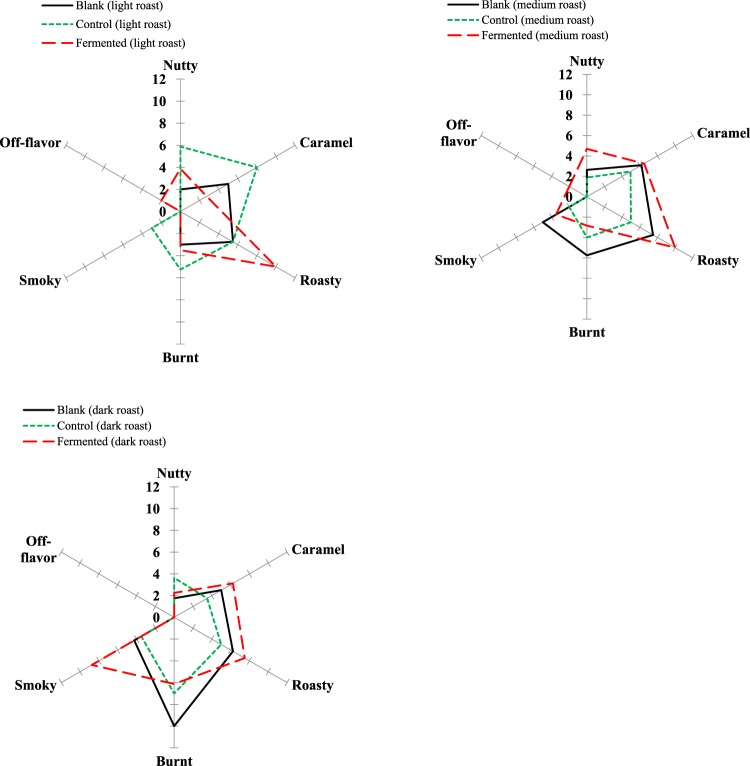
Aroma sensory profile of palm kernel oil (PKO) made from blank, control and fermented palm kernels (PK) with different roast degrees.

Sensory evaluation of PKO originating from different PKs were carried out to further characterize their distinct sensory properties. The results are shown in Fig. 8.

After light roast, the intensity of each sensory attribute in the fermented PKO is generally lower as compared with that of the control, except roasty and off-flavor. This is expected, as in comparison, fermented PKO contained fewer volatile compounds such as pyrazines, furans, furanones, pyraones and phenols, which generally represent nutty, caramelic and smoky odor. On the other hand, the fermented PKO had more free fatty acids, which could have contributed to a great extent to the off-flavor. This off-flavor perception in fermented PKO persisted to medium roast. Of medium roast, the levels of almost all volatile categories in fermented PKO caught up, making its each sensory attribute exceed that of the control but comparable to the blank, except the burnt note. Moreover, its higher levels of *N*-heterocyclic compounds help to impart it a more nutty and roasty characteristic. This phenomenon almost continued when roast proceeded to the dark level. In addition, after dark roast, the off-flavor in fermented PKO could not be detected although there was still a large amount of free fatty acids present. This is probably due to the masking of other heightened aroma attributes such as smoky and roasty.

## Conclusions

*Y. lipolytica* fermentation of PK modified the volatile profile of the obtained PKO after kernel roasting. Due to the robust proteolytic activity of *Y. lipolytica*, more free amino acids were released after fermentation, which impacted the formation of volatile compounds by influencing the Maillard reaction and Strecker degradation especially with intensified roast degrees. More Strecker aldehydes and *N*-heterocyclic compounds were formed in PKO derived from fermented PK after medium and dark roast, giving the obtained PKO a different volatile profile and hence distinguished sensory attributes. Besides, the catabolism of *Y. lipolytica* brought some special volatile compounds to PKO such as 2-phenylethanol. However, the lipases of *Y. lipolytica* aggravated the hydrolysis of fat and released a large amount of free fatty acids, which might have contributed to the off-flavor being perceived in the PKO. Furthermore, the free fatty acids may have rendered a more intense oxidation during heating and the oxidation products interfered with the Maillard reaction and hence impacting the generation of volatile compounds. Nevertheless, the approach of combining fermentation with roasting provides a novel way to modulate the volatile composition of PKO. However, optimization of this approach by monitoring fermentation degrees, enzyme activities, roasting levels, among others, should be conducted in future study with the aim to enhance the formation of preferable aroma compounds while reducing undesirable ones.
